# Book Reviews

**Published:** 1813-12

**Authors:** 


### CRITICAL ANALYSIS
### *OF RECENT PUBLICATIONS*
IN THE
### DIFFERENT BRANCHES OF PHYSIC, SURGERY, AND MEDICAL PHILOSOPHY.

### *Edinburgh Medical and Surgical Journal*, No. XXXV.

I. *Report on the State of Vaccination in certain Districts of India, and in the Isles of France and Bourbon.* By W. Scot, Surgeon.

THE successful progress of vaccination in India is well known to our readers, from the various reports on it from that country analysed in this Journal. Mr. Scot's report contains, not only evidence of the success of this practice in India, but a view of it in the Isles of France and Bourbon, where its efficacy has been equally conspicuous. The destruction by the small-pox in these islands, previous to the discovery or introduction of vaccination, was most extensive.

"The Isle of France contains about 6000 whites, 7000 free blacks and people of color, and from 60,000 to 70,000 slaves. In the latter class, class, there are thirteen males to six females. The Isle of Bourbon has probably fewer whites, but is reckoned to contain many more slaves. The small-pox has generally appeared in both islands at the same periods, excepting in 1811, when it was confined to the Mauritius. In the year 1756, the small-pox first appeared as an epidemic at the Mauritius, and was extremely fatal; the contagion was kept up for a year, and it is computed, that two died of every five who were attacked by it. In 1771, it appeared a second time, and was no less formidable, so that the island was almost depopulated.

"In 1792, when the population had greatly increased, a third epidemic appeared, which, at the lowest computation I have seen, destroyed more than 20,000 people. None of the inhabitants can speak of it now without horror; and the year after, a law was enacted, condemning the captain or surgeons of any vessel to death, who should make a false declaration of the state of health of his crew, in respect to the small-pox. In August 1803, the Ulysses, a slave-ship from Mozambique, arrived at the Mauritius, having the small-pox amongst the slaves. She was sent back to the Seychelles, and the vaccine virus having by that time been carried from India to the island, the people were roused by the recollection of former calamities, and after a few preliminary experiments, immense numbers were vaccinated, but with very little care or attention. Perhaps not one case in a hundred was examined during the progress of the disease. I make this estimate from knowing that no note was kept by any surgeon of his inoculations, and from the difficulty, or oftener the impossibility, of seeing our patients, when our utmost efforts were bent on keeping a perfect register.

"In July, 1811, some cases of small-pox were brought from Madagascar, on board the captured French frigates, and the contagion was established on shore; yet, from that period till the end of November, at which time I left the island, between forty and fifty cases only had occurred; and I have lately been assured that the contagion has long since entirely ceased. On the 13th of November I report to the superintending surgeon on the island, that 'we have a melancholy proof of the carelessness with which vaccination has been hitherto practised, in the numbers attacked by small-pox; of about fifty cases of that disease, seven have been believed to be vaccinated.'

"Two months before the introduction of the small-pox, I found it necessary to warn the inhabitants, that, from every information I could gain of the previous practice, I had no doubt that great numbers who were supposed to be duly vaccinated, were still in reality exposed to variolous infection. I gave them a distinct account of the ordinary progress of the disease, and urged every person to have their children and slaves reinoculated, in all cases where the course of the vaccine had not been attentively examined. Great numbers were accordingly subjected to this precautionary measure of a second inoculation; but I regret that the documents I have by me do not enable me to state the result with precision, and, on this subject, I wish to avoid any assertion founded merely on memory.

 "The

"The exertions of the French surgeons were but little seconded by the people, who were most interested in their success. It is almost incredible, that a people, styling themselves polished and civilized, should evince such apathy, not only where the lives of their children, but their pecuniary interests in their slaves, were concerned. In a report of the 20th of August, 1811, to the superintending surgeon, I mention this indifference of the people, even after the small-pox contagion had been introduced. 'Instead of punctually assembling the subjects for inoculation, at a convenient time and place for the surgeon, he has often the task of searching the habitations himself, and of coming at various times to catch the opportunity of finding them unemployed. The inoculation once performed, they pay no attention to the necessity of frequent inspection during the progress of the disease; they content themselves with thinking, when they think at all, that if the pustule has been genuine, it will leave a mark upon the arm; a most fallacious mode of judging, since other kinds of matter inoculated may do the same, independent of the frequency of marks made by tattooing.

"'The practice appears to have been so slovenly, that were a thousand cases to occur of small-pox after alleged vaccination, my faith in its efficacy would not be shaken.'

"Yet, under all these disadvantageous circumstances, the astonishing contrast already stated, between the progress of the contagion of small-pox in 1792 and 1811, at the Isle of France, must carry conviction to the most sceptical mind."

The efficacy of the dry crust, as suggested and practised by Mr. Bryce, receives the full sanction of Mr. Scot; and the evidence he produces goes very far to establish the practice.

"Mr. Bryce's directions for the employment of the dry crust to propagate the vaccine infection, I applied to practice, perhaps more extensively than most other practitioners. Having found it nearly or fully as successful as the recent virus, I resorted to it, on account of its great conveniency. I shall record one instance sufficiently conclusive on this head. During the existence of small-pox at the Isle of France, in September 1811, some slave-ships were seized at Port Louis, by one of our frigates. The slaves were landed, to the number of 359, and put into one large building; 39 of them had evidently had the small-pox. As no time was to be lost in a case of such urgent danger, I immediately inoculated 320 of them, with the matter of crust dissolved in water, there not being sufficient recent virus to be had for this purpose. I had previously taken measures to have always at hand a large supply of these crusts, to send to distant quarters, or to meet any emergency. Of these 320 inoculations, 174 took effect, and only 53 of the 146 failures took the disease on a second inoculation, which was still performed principally, though not entirely with the matter of crust. The other failures were tried again and again with recent virus; 84 resisted every attempt, and 9 were sent to hospital, of whom I cannot at this time give any account."

Much

Much is due to Mr. Scot for the exertions he has made to extend the blessings of Jenner's discovery to the Mauritius.

II. *Case of Periodical Day-Blindness.* By John Isbell, Surgeon.

This case of periodical blindness is concluded to be syphilitic, for it was cured by mercurial ointment rubbed on the thighs. The peculiarities of it are described in the following passage.

"Mr. ——— complained of pains in his limbs, with occasional loss of voluntary motion and sensation in the left arm and leg; the pains being most severe during the night, and felt more particularly in the central parts of the bones. The joints were also affected, but in a much less degree. Daily, between the hours of eleven and two, a total loss of sight came on, preceded by a severe pain of the forehead, but seated principally over the orbits. The attack was generally of half or a quarter of an hour's duration, sometimes returning three or four times within the said hours. He was, besides, now and then deprived of speech, but which seldom continued more than a minute or two. His hearing always remained perfect. His body was much emaciated, which had been gradually increasing for the last three years."

III. *On Oil of Turpentine, &c. in Epilepsy.* By Edward Percival, M.D.

In three cases of this intractable disease, in the Hospital for Incurables near Dublin, the oil of turpentine was employed in pretty considerable quantity, with the effect of mitigating the epileptic paroxysm, but without curing the disease. Though the turpentine failed as to an absolute cure, it certainly manifested considerable powers in epilepsy: it was given without producing any distressing symptoms on the stomach and bowels; on the contrary, it proved gratefully cordial, strengthening the powers of digestion, and gently promoting intestinal and renal action. Dr. Percival observes, that its only apparently specific action was on the uterine system, it being to appearance a certain emmenagogue. The shortest of the three cases we shall cite as further explanatory of the action of a new remedy, at least, of its novel application.

"Margaret Harrison, æt. 25, of middle stature, and plethoric habit, became subject to epilepsy eight years ago, from a sudden alarm. The paroxysms of her disorder occurred chiefly at night, when she had usually two or three mild fits, each enduring about a quarter of an hour. She was admitted to the Hospital for Incurables in the summer of 1812. During the four preceding years she had experienced no menstrual discharge. Her general health and strength have have appeared good, her appetite rather voracious, but her intellect has been obtused, approaching to fatuity.

"July 15, 1812.—I directed for her pills of myrrh, steel, and aloes, which, in the course of a few days, induced a return of the catamenia. Her understanding consequently improved, her conduct became more regular and amenable, yet her epileptic fits recurred without any mitigation. She commenced the terebinthinate mixture on the 4th of November, in the proportion of three drachms of ol. terebinth. to a pint of mint-water, which had an immediate effect in abating the frequency and duration of her fits. She became more lively, and for the first time replied to a query of mine, by saying that the medicine had done her great service.

"Until the 4th of December her fits of epilepsy had nearly disappeared, when, without any manifest cause, they began to recur in a mitigated and less frequent degree than formerly. I directed for her a mixture, with one ounce of oil of turpentine in a pint of mint-water, of which she took two table spoonfuls every fourth hour, with immediate and decided benefit. On the 29th of the same month it was reported to me that she had relapsed to her former epileptic habits; though her fits were somewhat less frequent, and certainly more gentle than before the use of the terebinthinate mixture.

"On the 31st of January, 1813, I directed a decoction of two drachms of the dried leaves of foxglove to be administered in divided doses, with as little interval as possible. The effect of this medicine was extreme nausea, vomiting, and subsequently purging, which continued for the space of eight or ten hours. The fits of epilepsy, however, began shortly to recur as before, with less violence than previous to the use of turpentine, but apparently unmitigated by the administration of digitalis. Her fatuity continued without interval or abatement."

### IV. *Case of Injury to the Fœtus, without the Mother being affected.* By William English, Surgeon.

The *facts* in this occurrence we will present to our readers, and leave them to judge for themselves.

"A lady in my neighbourhood, a month before being delivered of her second child, was standing upon a pair of steps, reaching something off the top of a chest of drawers, when she slipped, and fell backwards. In her fall, her back, about the middle of the sacrum, came in contact with the key of the room door, which was in the lock, and broke the key in two. She lay for some time insensible, but, when she recovered, was surprised to find that she had power to get up, and fright seemed to be the only suffering she had to complain of from so very serious an accident, excepting a slight soreness and stiffness of the whole back and neck, which continued until, and for a few days after, the child was born.

"There was nothing untoward happened during labour, which was of the class called lingering; but the child being large, may account for that circumstance. Soon after birth, a considerable cavity was observed in the child's back, situated about the middle of the sacrum.

sacrum. There had evidently been an extensive abscess, which was barely cicatrized at the bottom, and the skin and cellular substance was thickened and puckered all round the outer edge. For five or six days after the infant was born, there was a slight oozing of thin gummy matter from the sore, caused, I believe, by the friction of the clothes, but it soon healed firmly, and the child continues well. I had some fears that this case would end in spina bifida; however, the injury happily extended no further."

### V. *On the good Effects of Cold Applications to Ulcers.* By P. Johnson, Surgeon.

Mr. Johnson relates one case only, but speaks of his success as very general. This case being short, we shall give in his own words.

"On having joined my ship, about five weeks since, I found one of her company with five deep and high-edged ulcers, situated between two and seven inches above the patella, which affected him for *many* months; some of them healing, while others were suppurating. My predecessor had used every means that he could devise for their cure, with very little good effect. The man had no constitutional appearances of scrofula, though these ulcers most strongly appeared to partake of that disease. The discharge was ill-conditioned; the ulcers communicated often with each other, as easily ascertained on pressure, or by the probe. Having seen no written document of my predecessor's treatment, I immediately began with poultices of oatmeal, moistened with salt water, to be changed whenever they became dry, with a little lint underneath. From the 21st of January, I used the salt water, by applying a cloth and bandage continually kept wet, the water being every hour changed for more drawn up along-side, up to this day (Feb. 18th), a period of twenty-nine days, when the ulcers are healed, and the man capable of doing his duty. I have made use of no dressing between the cloth and sores. The cloths were rinsed three or four times a-day, consequently very clean."

Very few surgeons are unacquainted with Baynton's admirable method of curing ulcerated legs, though there are some, even in London, who either do not know, or do not feel its value. The constant application of cold water in his method, we have often thought to be serviceable, principally by carrying off the accumulated caloric, and keeping the limb in an under temperature. We should be glad to have this ascertained, and to know what is to be attributed to pressure, what to the absorption of an aqueous fluid, what to keeping the ulcer clean, and what to the abstraction of heat?

### VI. *On the external Application of Belladonna to the Eye, for the purpose of dilating the Pupil.* By T. Paget, Surgeon.

Mr. Paget, in this short paper, establishes his right to the first use of belladonna, in this country, for the purpose of dilating the pupil of the eye.

VII. *Observations on a Species of Vaginal Hernia occurring in Labour; read at a Meeting of the Medical Society of Liverpool.* By T. CHRISTIAN, Surgeon.

This paper describes a case which sometimes occurs in the practice of midwifery, and becomes important or hazardous only by being misunderstood. The bladder gets disturbed from its natural scite, and descends before the membranes in labour into the pelvis, obstructing the progress of the fœtus. The obvious remedy is emptying the bladder by the catheter.

VIII. *The Effects of cold Water given internally, or applied externally, in four Cases of Abdominal Inflammation.* By T. SMITH, Surgeon, &c.

We have long considered the depletion of heat, in all cases where temperature is much raised, to be one of the most efficacious remedies, especially in all cases of inflammation. Since this principle was urged in our Half-yearly Reports of the Progress of Medical Science, we have observed cold to have been applied with great boldness, and in some cases where heretofore the employment of heat had been thought to be beneficial.

The four cases here related are strongly in point. The 3d of these being short, and one of those in which the application of cold has been considered as peculiarly dangerous, we shall cite, as a specimen of Mr. Smith's practice.

"August 20th, 1812, I was called to the wife of Hugh Ross, carpenter, Dunaughton. She had been delivered of a child on the 15th, and on the 17th was seized with cold shivering, and pain in the belly and head, the lochia and secretion of milk being greatly diminished. I found her in the following condition:—respiration quick, oppressed, and suspiratory; pulse 150, extremely feeble, and at times intermitting. She complained of pain in her belly and forehead; her abdomen much tumefied, hot, and so tender that she could hardly bear it to be touched. Her attendants said that she was at times delirious, and that she had a second shivering of cold a short time before I arrived. She vomits frequently, and the fluid vomited is very acid; tongue dry and brown. She does not complain of thirst, but drinks with avidity when it is offered her. Hands and feet cold; lochia suppressed; mammæ flaccid. She is said to have had one or two loose stools to-day of a frothy appearance. The chalk mixture was given her, and cloths wet with cold water were desired to be applied over the whole abdomen.

"21st.—I saw her early this day. The cold cloths have been applied frequently, with much relief to the feelings of the patient. She has has had two or three loose stools; vomiting has ceased; appears less debilitated to-day; abdomen still tumid, hot, and tender; pulse 135.

"I now renewed the cold applications, which had been abandoned for some hours, applying cloths wet with cold water in which salt was dissolved, and renewing them as soon as they became hot. This practice was continued for about an hour, after which, upon examining the pulse, I found it 108 in the minute, and full. The patient expressed no uneasiness from the cold applications; on the contrary, she said they removed that sense of heat internally, which she had felt most distressing; and I observed, that after the cold water had been applied some time, the tenderness of the abdomen became much less, so that at length she could bear it to be firmly pressed without experiencing almost any pain.

"23d.—By message, I was informed to-day that the looseness continued with the effect of weakening her extremely, and that she had considerable cough and pain in the belly, aggravated by cold drinks, which she had taken by my directions. I sent some chalk powders, with orders to give one after every loose stool; and desired her drinks to be made warm.

"28th.—I was called to visit her to day. She had been much easier, though very weak since last report, till yesterday evening, when she was seized with a return of pain in the abdomen; pulse 128; tongue foul; thirst; cough; no stools.

"Applicetur abdomini emplast. mag. vesicator. et capiat omni bihorio, donec exoneretur alvus, Pulv. rhei, Pulv. glycyrrh. āā. gr. v. M.

"This patient residing at a considerable distance from me, I did not see her again till about three weeks after the date of the last report, when, upon calling, I found her on foot, with no complaint except occasional pains in the abdomen, which appeared to arise from costive bowels. She was now nursing her child, and had abundance of milk, By the occasional use of the pil. rhei comp. she was soon liberated from the pains, and now enjoys a good state of health."

### IX. *Case of Hæmatemesis.* By W. Cooke, Surgeon.

This is a minute diary of a case of some interest. The discharges of blood by vomiting, and per anum, were very large; and at length the patient sunk under them. Examination, *post mortem*, does not seem to have elucidated this case much: as our readers may possibly think differently, we insert it.

"The omentum and external surface of the stomach and duodenum were natural, except that the fat had an unusual yellow appearance. The jejunum, ilium, and colon, had a dark aspect, apparently in consequence of matter contained in them, by which they were much distended.

"The liver was very considerably enlarged, extending into the left hypochondrium, of a reddish color, and scirrhous throughout; the gall bladder was distended, the coats thickened, and the bile apparently black, but, in dilution with water, became yellowish green.

 "The

"The spleen was enlarged to double its natural size, resembling in firmness and color healthy liver.

"When the stomach was opened, it was found to contain only the sago taken a few hours before death. The villous coat seemed natural, except near the cardiac orifice, where there was a very circumscribed appearance of inflammation, and a more extensive one of ecchymosis. On the membrane of the œsophagus were numerous petechiæ. The duodenum contained thick yellowish matter. In the jejunum and ilium was a large quantity of dark matter, which in some parts was mixed with blood, and resembled black currant jelly. The colon contained a quantity of black offensive fæces.

"The villous coat of the intestines looked natural except in the colon, where it had a deep red color, not from increased vascularity, but ecchymosis.

"The pelvis of the left kidney contained some pus. The bladder was natural. The urine (of which there was about half a pint), had a sediment of thick matter, probably from the left kidney.

"The blood in the mesenteric veins was so hard as to give them the feel and appearance of injected vessels. The lungs were of a healthy color, the left a little hardened; and some yellow lymph was deposited beneath the pleura pulmonalis.

"The cavity of the chest, and the pericardium, contained more fluid than natural to them.

"On cutting into the lungs, the cells appeared full of a *sero-mucous* fluid, which had given to the left its unnatural solidity.

"The heart and vessels were natural."

X. *Pathological and Practical Observations.*

This paper being only in part published, we postpone our account of it, until the whole is before us.

XI. *Severe Affection of the Stomach, ascribed to the presence in it of an Animal of the Lacerta tribe.* By JOHN SPENCE, M.D.

This is one of those extraordinary occurrences which require most positive demonstrative evidence, to give it currency. A stout country girl, 21 years of age, has serious derangement of the functions of the stomach and intestines for several days. On the 17th of December, in the night-time, after having taken some strong doses of calomel, and a large solution of neutral salts, she passed a reptile of the Lacerta species. This, however, Dr. Spence did not see, but gives the account from an old woman and the girl, who did both of them see it. Dr. Spence says,

"I was vexed, and much disappointed, at not being able to procure the animal alive or dead; for, although I can have no doubt of the fact myself, yet neither my testimony, nor that of the people themselves, will be sufficient to satisfy the incredulity of others. I, however, made such inquiries, that there could be no deception. When at stool, she had unusual pain in the rectum, and afterwards she thought she perceived something moving in the pot. After examining with a stick, it leaped out with a bound, and ran very nimbly under the drawers, which put both her and her mother, who was in bed in the room, into great fright and consternation. This they saw by the light of the fire. She next lighted a candle, and, in looking under the drawers, was still more frightened, when she saw the animal with staring clear eyes. By the intreaties of her mother, who was not able to get out of bed herself, and was afraid it should do mischief, she laid hold of it when it turned round, and put it in the fire, and held it down with the poker till it was consumed. It squeaked with a shrill noise, and attempted to get out when first put in the fire.

"The size of the animal, as she described it by comparison with her finger, was between four and five inches long, and considerably thicker than the finger; it had a bluff nose, like the end of the finger, with a considerable mouth and bright staring eyes; the back, of a mahogany color, with a number of small white bright spots; had four feet with claws, not very long; and a short thick flattened tail, about an inch long. Did not take notice of the belly."

Though Dr. Spence believes this relation himself, he justly observes the evidence will not be sufficient to satisfy the incredulity of others. We are among the incredulous, and are much more disposed to believe this lizard to have been only in the pot, and not in the girl's bowels.

XII. *An Account of some Cases of Puerperal Fever, with their Treatment.* By T. SUTTON, M.D.

Though there may be some doubt whether these cases may have been what every practitioner would call puerperal fever, yet there can be no hesitation as to their hazardous nature, and their being connected with local inflammation. The application of cold to the abdominal parietes was most evidently beneficial.

XIII. *Case of Scirrhus in the Intestines, arising from Hairs remaining in the Canal.* By W. G. BURRELL, M.D.

After a variety of dyspeptic symptoms, constipation, and irregular action in the intestinal canal, the patient, a soldier 35 years of age, apparently worn down by irritation, expired in May 1812. The examination of the body after death, was supposed to ascertain the cause of the morbid actions which had so long afflicted the patient.

"On laying open the abdomen, the stomach was found much thickened throughout its whole substance, and the pylorus very much contracted, which contraction continued down the duodenum. Through all the intestines this thickening and gristly appearance was apparent. The colon was prodigiously enlarged in its calibre until where it forms its sigmoid flexion. At that point there were three distinct holes ulcerated through the coats of the intestines, and forming a communication with the abdominal cavity.

"Beyond the sigmoid flexion the intestine was contracted in its diameter, so as hardly to admit the little finger to pass downwards.

"On cutting open the pylorus and small intestines, the internal coats were found to be covered with a soft substance, which resembled size. The internal coats of the colon were of a dark color, and in general were ulcerated completely, and were hanging in shreds. The color of the colon was of a dark lurid red. At the sigmoid flexure there was much contraction, and the thickening was so great on one side, and the valve found so considerable, as hardly to admit a common bougie through it.

"The portion forming the sigmoid flexure was cut out; and, on laying it open, and removing some hardened portions of fæces, five or six hog's bristles were seen distinctly crossing each other in different directions, and were partially invested in the villous coat, which had grown over them, and had retained them in the different positions in which they were placed; and so firmly were they kept down by those partial coverings, that it required some force to draw them out. The mesenteric glands were of a cartilaginous appearance; the liver was suffused with blood, and the gall-bladder full of bile.

"The spleen was very small, and compressed into an oblong shape, probably arising from the pressure of the colon when distended with feculent matter.

"This man had formerly been a shoemaker. There was no certainty at what period he swallowed those hairs; but from the derangement which always existed in his bowels, and the pain referred to the situation of the sigmoid flexure, little doubt can be entertained but that these hairs were the cause of all his complaints, and ultimately of his death."

XIV. *Account of the second Watch of the reputed Fasting Woman.* By B. Granger, Surgeon, &c.

Mr. Granger, who had for a long period attended to the case of Ann Moore, gives here a detail of occurrences during the investigation, which ended in the detection of this woman's imposition.

XV. *Observations on Brain Fever.* By S. B. Pearson, M.D.

This is a reprint of a small pamphlet published by Dr. Pearson, on this fever. Some further remarks on this disease are added by Dr. Pearson, corroborative of his practice, and directions for the treatment, founded on principle and supported by experience.

XVI. *A Mode of preventing the shortening of the Limb, in conducting the Cure of the broken Femur.*

This projector proposes to suspend the nates in such a manner that their weight may keep the fractured thigh constantly on the stretch. How far a patient with a fractured femur can bear this, experiment must determine.

